# Targeted Monitoring on Illegal Suid Meat and Meat Products Trade in Italy: African Swine Fever Detection and Implications for Transboundary Spread

**DOI:** 10.1155/tbed/4119953

**Published:** 2025-11-21

**Authors:** Silvia Pavone, Roberta Biccheri, Maria Serena Beato, Carmen Iscaro, Clara Montagnin, Cristina Casciari, Giulia Costantino, Cecilia Righi, Claudia Torresi, Stefano Petrini, Paola De Santis, Bianca Maria Varcasia, Monica Giammarioli, Luigi Ruocco, Nicola Santini, Vincenzo Caputo, Francesco Feliziani

**Affiliations:** ^1^National Reference Laboratory for Pestivirus and Asfivirus, Istituto Zooprofilattico Sperimentale dell'Umbria e delle Marche “Togo Rosati” (IZSUM), Perugia, Umbria, Italy; ^2^Istituto Zooprofilattico Sperimentale del Lazio e della Toscana, Roma, Lazio, Italy; ^3^Ministero della Salute, Roma, Lazio, Italy

**Keywords:** African swine fever virus, epidemiology, illegal import, Italy, meat products, surveillance, transboundary transmission

## Abstract

African swine fever (ASF) is one of the most threatening animal diseases for the global swine industry. Due to the high risk of ASF virus (ASFv) transmission via infected suid meat and derived products, Italy adopted specific risk mitigation measures during 2023 and 2024. Two targeted programs were established to implement these measures: one addressing the illegal domestic trade of wild boar meat and meat products and the second focusing on irregularities in food imports. In both programs, products with traceability irregularity and improper or missing labels were seized. ASFv detection by real-time PCR was conducted on all suid meat and derived products originating from illegal national trade, as well as on illegally imported products in which suid DNA was detected. While smuggled local products did not show any ASFv contamination, a large proportion of the illegally imported products tested positive for ASFv by real-time PCR. However, experimental infection tests conducted both in vitro and in vivo using samples that tested positive by real-time PCR, yielded negative results indicating that the virus was inactivated. These programs highlighted the existence of an illegal network responsible for smuggling suid meat and derived products into Italy. Although in vivo and in vitro testing excluded the presence of infectious virus in illegally imported products, the potential risk of transboundary transmission through illegal importation remains a significant concern, necessitating ongoing surveillance and stringent biosecurity measures.

## 1. Introduction

African swine fever (ASF) is a nonzoonotic viral disease of suids caused by ASF virus (ASFv), recently reclassified as *Asfivirus haemorrhagiae* [1]. It represents one of the most significant animal health threats to the global swine industry [2–5]. The disease was first described in Kenya in 1921 [6]. Genotype I ASFv was introduced into Europe in 1957, triggering the first European epidemic wave [3]. While ASF was successfully eradicated from the majority of European affected countries, Sardinia region, in Italy, remained an exception, with the virus persisting since 1978 [7] until its recent eradication in September 2024. In 2007, ASFv Genotype II reached Georgia, affecting both domestic pigs and wild boars [8]. This event marked the beginning of a major epidemic worldwide, affecting over 50 countries [9–24]. Genotype II ASFv was first detected in the European Union in 2014 [16] and reached mainland Italy at the end of December 2021 [25].

By May 31, 2024, marking the conclusion of the monitoring programs described in this study, Italy registered 2192 positive cases in wild boars and 26 outbreaks in domestic pigs. At the time of writing, Italy counted three active infection clusters caused by the Genotype II ASFv. The so called “northwest cluster” includes an extensive infection area covering five Italian regions (Piedmont, Liguria, Lombardy, Emilia-Romagna, and Tuscany), primarily affecting wild boars, with seasonal outbreaks in domestic pigs [24]. Another cluster includes cases in two regions (Campania and Basilicata) [26], where ASFv was detected in a limited area but continues to show new cases. Additionally, a third cluster in Calabria region registered cases in wild and domestic pigs. A fourth cluster, located in central Italy (Rome and Lazio region), was officially eradicated in January 2025. This cluster was characterized by positive cases in both domestic and wild boar. While ASFv was promptly eradicated in domestic pigs, the wild boar cluster in Rome experienced alternating phases of epidemiological silence and epidemic resurgence [13, 26], which persisted until June 2024, when the last case in wild boar was notified.

ASFv spreads by contact with infectious animals and fomites, ingestion of contaminated pig products and, in areas where the biological vector is present, via tick bites [27]. The mechanisms underlying ASFv replication and dissemination within the host have been extensively investigated in experimentally infected domestic pigs [28]. Monocytes and macrophages are the main target cells of ASFv [29] and organs rich in cells of the mononuclear phagocyte system (MPS)—such as the spleen, lymph nodes, and bone marrow—as well as the liver, lungs, and kidneys, are the main sites of high viral replication [28]. The virus reaches these target sites via the lymphatic and circulatory systems, either free in plasma, bound to erythrocytes, or carried by infected monocytes [28]. In tissues containing few MPS components—such as the central nervous system, stomach, or large intestine—viral titers are generally attributable to the presence of virus in the blood, rather than to local replication [28]. However, in the later stages of infection, several other cell types, including endothelial cells, pericytes, and fibroblasts, may also become infected [30]. This broad cellular tropism supports the classification of ASF as a pantropic disease [28], with virtually all tissues in infected animals potentially harboring the virus. A recent study demonstrated that striated muscles, such as those from the shoulder, back, and thigh, contain ASFv genome copy numbers comparable to those detected in skin tissue, but lower than those observed in blood, spleen, and bone marrow—the primary target matrices [31]. The presence of ASFv in the muscle tissue of infected animals has direct implications for disease transmission. Given its remarkable stability across a wide range of temperatures and pH values, as well as its resistance to autolytic degradation, meat and pork-derived products from infected animals represent highly efficient vehicles for viral dissemination [4, 32–34]. This facilitates not only local transmission but also long-distance spread of the disease through human-mediated movement across regions and countries [35–38].

ASFv circulation in wild boars and domestic pigs in Italy poses a continuous threat to the swine industry and national economy, underscoring the critical need for effective control and monitoring strategies to prevent further spread. Since the first detection of ASFv Genotype II in Italy in 2021, several monitoring initiatives have been implemented to mitigate the risks associated with the illegal trade of contaminated swine meat products. Following a case of illegal importation of non-EU animal products into Italy, coupled with concurrent ASFv detection, it became evident that particular attention must also be paid to the importation of pork-based food products.

The aim of this study was to describe Italy's efforts to safeguard the swine industry through the implementation of two monitoring programs: one addressing the illegal domestic trade of wild boar meat and meat products and the other targeting the illegal importation of ethnic food. Both programs aimed to assess the potential risk these products pose for the introduction of ASFv in the country. The activities of both programs were supported by in vitro and in vivo testing to detect the presence of ASFv and evaluate its infectivity in confiscated products.

## 2. Materials and Methods

### 2.1. Monitoring Program for Illegal Domestic Trade of Wild Boar Meat and Meat Products

The Italian Ministry of Health instructed local veterinary services to intensify surveillance and control measures to ensure the compliance of meat and meat products from wild boars with trade regulations [39]. The program in force from September 1, 2023 to May 31, 2024 scheduled monitoring activities at local markets, fairs, agritourisms, public catering facilities, pork meat products processing establishments, ports, and airports. In case of traceability irregularity, meat or meat products were sampled and tested for ASFv and immediately destroyed. These control activities followed a standardized and nationally coordinated workflow. Local health authorities were responsible for sampling of meat or meat products irregularly traced. The seized samples were then forwarded directly to the National Reference Laboratory (NRL) for Swine Fever for ASFv detection using real-time PCR. All samples, together with their associated metadata, were registered in the laboratory's internal information management system.

### 2.2. Monitoring Program for Ethnic Food Import

A national monitoring program for imported ethnic foods was implemented [40]. This program, in force from December 22, 2023 to May 31, 2024, foresaw at least 30 inspection activities per region and 15 for the two autonomous provinces conducted at city shops, stores, and rural markets, aimed at identifying any illegally imported products. When a product was found to be improperly labeled or unlabeled, in violation of regulation on the provision of food information to consumers (Regulation (EU) Number 1169/2011) [41], it was seized by the local health authorities and submitted for swine species identification testing, either at the territorially competent Istituto Zooprofilattico Sperimentale (IZS), if the test was available, or at the nearest IZS equipped to perform the analysis. Products that tested positive for swine origin and showed no evidence of inactivation treatment, as defined in Annex VII of Commission Delegated Regulation (EU) 2020/687 [42], were forwarded to the NRL for ASFv detection. Similarly, when uncertainty existed regarding the information provided on packaging or labels about such treatment, the precautionary principle was applied and the products were also submitted to the NRL for testing. In all cases, noncompliant products were confiscated from the operators.

### 2.3. Metadata Collection of Confiscated Products

For each confiscated sample, the competent authority completed a sampling report containing key metadata relevant for product identification, classification, and epidemiological context. The collected information included:

– Date of collection.
– Geographic coordinates of the point of sale.
– Type of point of sale (e.g., supermarket, grocery store, corner shop, delicatessen, ethnic food store, agritourism, etc.).
– Product type and list of ingredients as reported on the label (when available). Labels written in foreign languages were translated into Italian using software tools for language recognition and translation.
– Evidence of inactivation treatment (or absence thereof), as defined in Annex VII of Regulation (EU) 687/2020 [42].
– Origin of the confiscated product (local trade, following the monitoring program for illegal domestic trade of wild boar meat and meat products, or import, following the monitoring program for ethnic food import).

Based on this information, the product category (e.g., compound food, meat product, meat preparation, and meat) as defined by current legislations [43, 44], and the processing category, according to the NOVA classification system, which classifies foods as unprocessed or minimally processed (Group 1), processed culinary ingredients (Group 2), processed foods (Group 3), and ultraprocessed foods (Group 4) [45], were determined only for products that tested positive for ASFv by real-time PCR. This was done to assess whether product category and processing level could be associated with viral persistence and the preservation of infectivity.

### 2.4. Laboratory Analysis

#### 2.4.1. Microarray for Species Identification

Genetic analysis for the detection of swine genetic markers (species identification) were performed at the Istituti Zooprofilattici Sperimentali (II.ZZ.SS.), that are the official laboratories in charge for one or two Italian regions. Here, we report the most commonly used method. Total DNA was extracted and purified using the DNeasy mericon Food kit (Qiagen, Italy), following the manufacturer's instructions. In brief, lysis buffer and proteinase K were added to 200 mg of homogenized food and incubated for 30 min at 60°C, with constant shaking. After incubation, the sample was cooled on ice to room temperature and centrifuged at 2500 × *g* for 5 min. The clear supernatant (~700 µL) was mixed with 500 µL of chloroform, vortexed for 15 s, and centrifuged at 14,000 × *g* for 15 min. Approximately 350 µL of the aqueous phase was mixed with 350 µL of PB buffer (Qiagen, Italy), transferred to a spin column, and centrifuged at 17,900 × *g* for 1 min. To remove inhibitors, the column was washed with 500 µL of AW2 buffer, centrifuged at 17,900 × *g* for 1 min, and the effluent discarded. Residual ethanol from the AW2 buffer was removed by a final centrifugation at 17,900 × *g* for 1 min to dry the membrane. DNA was eluted from the column by adding 100 µL of elution buffer. The extracted DNA was suitable for microarray analysis, and its concentration was determined using NanodropOne (Thermo Fisher Scientific Inc, Italy); the final concentration was defined as 20 ng/µL.

The microarray assay was performed using a commercial kit (GeneTop Meat kit, Lifeline Lab, Italy), which included four steps: (i) DNA amplification; (ii) amplicon denaturation; (iii) amplicon hybridization; (iv) reading and interpretation of the results. All the reagents for the microarray test including positive controls for DNA amplification, were provided in the GeneTop Meat kit (Lifeline Lab, Italy).

DNA amplification was carried out according to the manufacturer's protocol. The master mix (22.25 µL) and Taq polymerase (0.25 µL) were combined and 22.5 µL of the mix was added to each PCR tube, along with 2.5 µL of template DNA. Thermal cycling conditions were as follows: initial denaturation at 94°C for 5 min; 30 cycles of denaturation at 94°C for 30 s, annealing at 60°C for 30 s, and extension at 72°C for 30 s; followed by a final extension at 72°C for 10 min. For each batch of samples analyzed, a negative control and two microarray positive controls (meat chip positive control (A/B)) were included.

Hybridization was carried out using a hybridization oven (Thermo-Shaker PST-60HL, Biosan, Italy) 50 ± 1°C for 40 min, with continuous stirring at 1000 rpm. A 100 µL aliquot of the hybridization buffer (HA Buffer, GeneTop meat kit, LifeLineLab, Italy) was added to an adequate number of wells in the strips supplied with the kit. Each well was coated with the probes for the various animal species to be identified. Before the hybridization, the amplicons were denatured at 95°C for 5 min and 30 s, then immediately cooled on ice. A total of 5 µL of each denatured amplicon was added into the corresponding well. At the end of hybridization, the wells were washed three times using about 200 µL of wash buffer for each washing step.

Successful hybridization detection, indicating specific probe-target binding, was achieved using streptavidin conjugated with alkaline phosphatase and subsequent staining with NBT/BCIP. Results were interpreted based on the formation of blue–violet spots that follow a specific arrangement within the well for each species detected, as indicated in the reading diagram provided in the GeneTop Meat kit (LifeLineLab, Italy). The test is considered valid only if all controls (hybridization and positive and negative PCR controls) were consistent.

#### 2.4.2. ASFV Detection by Real-Time PCR

The samples received by NRL were homogenized to obtain a 10% *w*/*v* suspension, which was then centrifuged at 1200 × *g* for 10 min in the Biosafety Level III laboratory. DNA was subsequently extracted using the High Pure PCR Template Preparation Kit (Roche Diagnostics GmbH, Roche Applied Science, Mannheim, Germany) following the manufacturer's instructions. The NRL performed real-time PCR assays as described in the World Organization for Animal Health (WOAH) manual [46], based on the protocols reported by King et al. [47] targeting a conserved region of the VP72 gene. The method was validated for use in food matrices (unpublished data) and the assays were conducted under the UNE-EN ISO/IEC 17025:2005 guidelines [48].

#### 2.4.3. Molecular Characterization

The samples were genotyped by partial Sanger sequencing of a fragment of the B646L/p72 gene, as previously described by Agüero et al. [49]. The central variable region (CVR), within the B602L gene, was also analyzed. For Genotype I, the CVR was amplified using the primer pair ORF9L-F/ORF9L-R, which flanks a DNA fragment of variable length [50, 51]. For a specific amplification of CVR in ASFv Genotype II, the primer pair CVR1 and CVR2 was used, as reported by Gallardo et al. [52].

After gel electrophoresis, PCR products were purified using an ExS-PURE PCR cleanup Kit (NimaGen BV, Nijmegen, The Netherlands). Both the sense and antisense strands were sequenced using Sanger sequencing with three independent reactions performed for each isolate. Sequencing was carried out using the BrilliantDye v1.1 cycle sequencing Kit (NimaGen BV, Nijmegen, The Netherlands), and the dye terminators were removed with the DyeEx 2.0 Spin Kit (Qiagen, Hilden, Germany). Sequencing products were were analyzed using an ABI PRISM 3130 Genetic Analyzer (Thermo Fisher Scientific, Waltham, MA, USA), and the resulting sequence data were processed with SeqMan Pro program Version 15.0 (DNASTAR, Madison, WI, USA) [53].

The B646L/p72 sequence dataset was analyzed, and the nucleotide sequences were aligned to the published ASFv reference strains retrieved from GenBank using Clustal X.2 [54], and manually cured using BioEdit software (version 7.0) [55]. Phylogenetic tree was inferred using the maximum likelihood (ML) method in MEGA 11 [56]. Cluster robustness was evaluated with 10,000 bootstrap replicates, and bootstrap values greater than 70% were reported. All nucleotide sequences generated in this study are available in GenBank [57].

#### 2.4.4. Virus Isolation in Cell Cultures

Virus isolation attempts were carried out on all real-time PCR positive samples. In brief, primary leucocyte cultures were obtained from swine peripheral blood mononuclear cells (PBMCs) collected from healthy large white outbred pigs typically aged between eight and twelve weeks housed in registered farm, under the approval of the Italian Ministry of Health (Authorization Number 931/2029-PR) and in accordance with European legislation on the protection of animals used for scientific purposes [58]. Preparation of PBMC involved processing unclotted fresh blood following the procedure outlined in the Manual of Diagnostic Tests and Vaccines for Terrestrial Animals (WOAH) [46]. After a 3-day culture period, PBMC were infected at a 1:10 dilution with the PCR-positive samples. The infected cells were then incubated at 37°C in a 5% CO_2_ environment for 7 days in the presence of red blood cells. Plates were observed over the course of 7 days to detect the presence of hemadsorption (HAD). Titration of the ASFv was performed on PBMC to determine the endpoint dilution, and viral titer was assessed as the amount of virus causing HAD in 50% of infected cultures (HAD50/mL) [59]. In the absence of characteristic “rosettes” attributable to ASFv, two additional passages on PBMC were performed to reach a definitive negative result. If the samples did not exhibit HAD, but showed cytopathic effects (CPEs), further investigation for the presence of non-HAD strains was conducted by real-time PCR [47] and additional passages in PBMC.

#### 2.4.5. Animal Experiments

An in vivo experiment was conducted to further investigate the ASFv infectivity of real-time PCR positive samples. Four crossbreed pigs of approximately 25 kg each, clinically healthy and ASFv-free, were housed in the BSL3 animal facilities at the NRL. Pigs had access to water and feed ad libitum. All care and experimental procedures were established according to the European legislation on the protection of animals used for scientific purposes [58]. The experiments were carried out under the approval of the Animal Welfare and Experimentation Committee of IZSUM and by Italian Ministry of Health (Authorization Number 143/2023-PR). Prior to the start of the experiment, the pigs were acclimated for 7 days. Thirty real-time PCR-positive samples were randomly selected, ensuring variability in product type, for use in the experimental trial (Table S1). In detail, the four pigs were divided into two groups of two pigs each: Group 1 was fed with 15 ASFv PCR-positive food samples with a cycle threshold (Ct) ≤ 30 by real-time PCR; Group 2 was fed with 15 contaminated food samples with a Ct > 30. All pigs were monitored daily for clinical signs and rectal temperature for 19 days postfeeding (DPF). EDTA blood and serum samples were collected for each animal on Days −1 and 19 DPF. The blood samples were used to detect viremia by real-time PCR, following the protocol described above. Serum samples were tested for antibodies against ASFv using the competitive ELISA ID Screen African Swine Fever Competition kit (ID.VET, rue Louise Pasteur—Grabels, France), following the manufacturer's instructions. Optical densities were read with an automatic plate reader (Sunrise; Tecan Infinite F50, Männedorf, Switzerland), and data were analyzed using Magellan software version 7.1 (Tecan, Männedorf, Switzerland). At the conclusion of the trial, all animals were culled and subjected to a full necropsy. Spleen samples were collected from each pig and then tested by real-time PCR assay [47].

## 3. Results

Between September 1, 2023 and May 31, 2024, a total of 442 confiscated samples were collected and submitted to the NRL for ASFv detection as part of monitoring programs targeting both domestic trade and food importation. Of these, 58 samples were seized and tested under the program for illegal domestic trade of wild boar meat and meat products, while 384 samples were analyzed within the monitoring program for ethnic food imports. These latter samples were characterized by improper or missing labeling, tested positive for suid DNA through genetic analysis targeting swine-genetic markers, and showed no evidence of inactivation treatment, as defined in Annex VII of Regulation (EU) 687/2020 [42].

**Figure 1:**
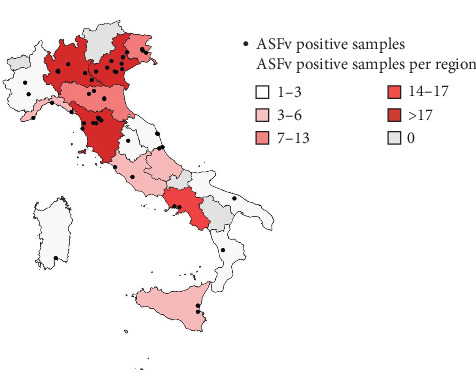
Regional distribution of ASFv-PCR-positive samples detected during the monitoring program for illegally imported ethnic food. The color gradient of each Italian region indicates the total number of PCR-positive samples identified within that region, while black dots represent the sampling locations where ASF-PCR-positive samples were collected.

Among the analyzed samples, 26% (114/442) tested positive for ASFv by real-time PCR, while 72% (321/442) tested negative, 1% (5/442) yielded inconclusive results, and 1% (2/442) were deemed unsuitable for testing. All ASFv-PCR-positive samples originated only from monitoring program for ethnic food imports, whereas none from monitoring program for illegal domestic trade of wild boar meat and meat tested positive by real-time PCR. The spatial distribution of the PCR-positive samples is represented in Figure 1. These samples were not evenly distributed: the highest numbers were detected in two northern regions of Italy (Veneto and Lombardy) and one central region (Tuscany). Overall, the northern regions exhibited the greatest number of irregular points of sale and ASFv-PCR-positive samples, associated with deficiencies in product traceability and labeling. Certain points of sale were repeatedly sampled during the monitoring program, and multiple illegal products were sometimes collected from the same location, either during a single inspection or across different inspections.

Among all ASFv-PCR-positive samples, 22% (26/114) showed no evidence of inactivation treatment, as defined in Annex VII of Regulation (EU) 687/2020 [42]. For the remaining samples, the local veterinary services reported uncertainty regarding the application of such processes (Table S1). The ASFv-PCR-positive samples comprised 69% (79/114) compound foods, 20% (23/114) meat preparations, 12.7% (10/114) meat products, and 2% (2/114) meat (Table S1). The origin of the illegally imported products was generally unspecified, and the packaging labels were predominantly written in Chinese. According to the NOVA classification system [45], 97% (113/114) of the products were classified as Group 4, while only 1% (1/114) belonged to Group 3 (Table S1). Specifically, the single Group 3 product consisted of dried meat. Among the Group 4 products, two were raw meats prepared with culinary ingredients (sugar, oil), food additives (monosodium glutamate), and processed foods (soy sauce).

Virus isolation attempts from the 114 ASFv-PCR-positive samples yielded negative results. All pigs that were fed with ASFv-PCR-positive products remained clinically healthy, showing neither fever nor other signs of disease throughout the 19 DPF observation period. Postmortem examinations revealed no lesions suggestive of ASFv infection, and both blood collected at Days −1 and 19 DPF as well as spleen samples at necropsy tested negative for ASFv by real-time PCR. Similarly, serological analysis of sera sampled at Day −1 and prior to culling also yielded negative results.

A total of 80 ASF partial B646L/p72 sequences were successfully generated and aligned with reference strains of ASF Genotypes I, II, III, V, VIII, IXX, XX, and XV available in GenBank [57]. All 80 samples generated a 257 bp fragment sequence belonging to the ASFv. Reliable sequences could not be obtained from 35 samples due to poor DNA preservation.

**Figure 2:**
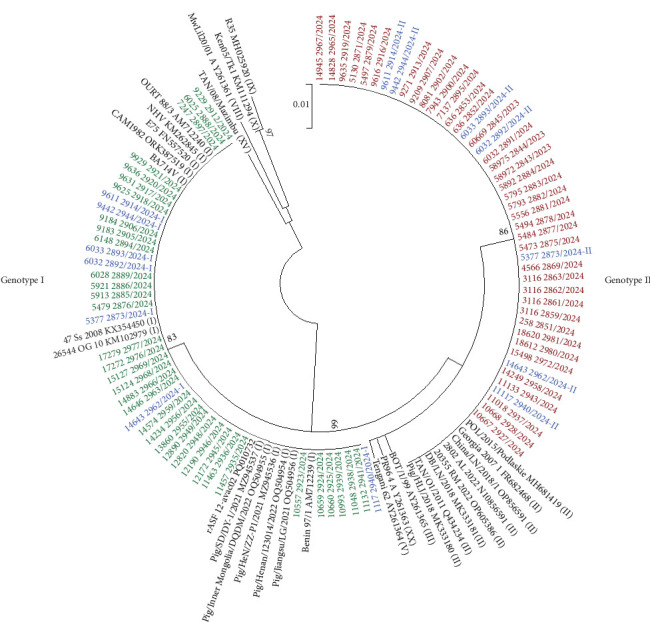
Phylogenetic tree constructed using 257 nt from the partial B646L gene of 87 ASFv PCR-positive samples collected from smuggled food and reference strains retrieved from the GenBank database. The numbers on the major nodes indicate the bootstrap values in percentage, obtained from 10,000 replicates. Bar: number of substitutions per site. Newly characterized samples are reported in colors. Genotype I samples are marked in green, Genotype II samples are marked in red, while the Genotype I/II are marked in blue. At nucleotide positions 18, 60, 207, and 231, base-calling revealed equal signal intensity for two nucleotides, leading to the identification of mixed consensus sequences. One consensus was characterized by SNPs typical of Genotype I, the other by SNPs characteristic of Genotype II.

Among the 80 partial sequences, 42 were classified as ASFv Genotype I, while 45 clustered with Genotype II. The list of accession numbers, along with the genetic characteristics of ASFv sequences obtained from each food sample is provided in Table S1. In seven samples, the partial B646L/p72 sequences obtained (11117_2940/2024-PV667735/PV667688; 14643_2962/2024-PV667746/PV667691; 5377_2873/2024-PV667753/PV667704; 6032_2892/2024-PV667759/PV667716; 6033_2893/2024-PV667760/PV667717; 9442_2944/2024-PV667766/PV667726; 9611_2914/2024-PV667767/PV667727) showed four SNPs, confirming the simultaneous presence of both genotypes, I and II (Figure 2).

Specifically, the partial B646L/p72 sequences clustering within Genotype I, showed at position 18 the substitution of a T with a C, at position 60 the substitution of a G with a T, in position 207 the substitution of a T by a C and finally in position 231 the substitution of an A by a G, compared to Genotype II. None of these substitutions altered the amino acid sequence.

Sequence identity within the same genotype was 100%. Cross-genotype identity between Genotypes I and II sequences was 98.4%.

To further investigate intragenotypic diversity among the seven dual-genotype samples, additional sequencing was performed on the CVR of the B602L gene. These sequences were compared to published ASFv CVR sequences from GenBank [57], representing known variants for Genotypes I and II (Table S1) [52, 60].

**Figure 3:**
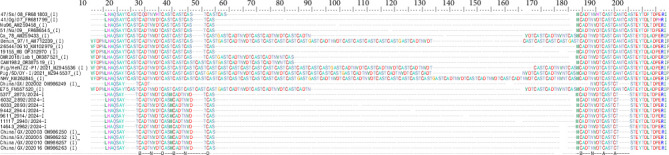
Amino acid sequence alignment of the tetramer tandem repeats identified within the central variable region of gene B602L (CVR) from smuggled ASFv PCR-positive Genotype I samples (BNDBNDBNAA). Key: A, CAST; a, CVST, CTST, and CASI; B, CADT and CTDT; C, GAST and GANT; D, CASM; F, CANT; N, NVDT and NVGT; T, NVNT; H, RAST; S, SAST; O, NANI, NADI, and NASI; V, NAST, NAVT, NANT, and NADT. Dashes indicate gaps introduced to enable similarities between sequences to be more easily visualized.

The CVR sequences from Genotype I isolates yielded 178 nucleotide-long amplicons. After translation into amino acids, phylogenetic analysis placed the seven Genotype I viruses within the Tet-10a variant group, alongside strains previously identified in southern China (Figure 3).

**Figure 4:**
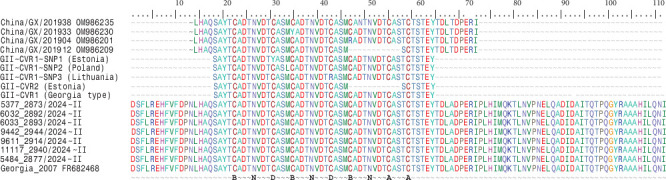
Amino acid sequence alignment of the tetramer tandem repeats identified within the central variable region of gene B602L (CVR) from smuggled ASFv PCR-positive Genotype II (BNDBNDBNAA). Key: A, CAST; a, CVST, CTST, and CASI; B, CADT and CTDT; C, GAST and GANT; D, CASM; F, CANT; N, NVDT and NVGT; T, NVNT; H, RAST; S, SAST; O, NANI, NADI, and NASI; V, NAST, NAVT, NANT, and NADT. Dashes indicate gaps introduced to enable similarities between sequences to be more easily visualized.

Amino acid analysis of the CVR tetramer repeats revealed a consistent motif of 10 tetramers, with the repeat pattern BNDBNDBNAA. This profile matches those reported for ASFv strains circulating in southern China during 2019–2020 [61] and was detected in both Genotype I as the most dominant variant. In contrast, Genotype II CVR sequences from the same seven samples yielded a 455 bp amplicon. These sequences grouped within Genotype II, Subgroup I, along with the reference strain Georgia 2007/1 [52] (Figure 4).

However, phylogenetic analysis confirmed that these sequences do not represent a novel subgroup. Instead, they corresponded to the previously described CVR Genotype II-variant I (CVR1), which also displayed the characteristic BNDBNDBNAA tetrameric sequence (Key: A, CAST; a, CVST, CTST, and CASI; B, CADT and CTDT; C, GAST and GANT; D, CASM; F, CANT; N, NVDT and NVGT; T, NVNT; H, RAST; S, SAST; O, NANI, NADI, and NASI; V, NAST, NAVT, NANT, and NADT).

## 4. Discussion

Pig farming is a fundamental component of Italy's livestock sector, deeply rooted in the country's historical, cultural, and economic fabric. The cured pig meats industry alone generates over 8 billion euros, accounting for approximately 5.6% of the national food sector [62]. Italy produces around nine million pigs annually, with production concentrated in northern regions, such as Lombardy and Emilia-Romagna, renowned for their high-quality pork products [62]. The country is home to numerous protected geographical indication (PGI) cured meats, such as Cotechino Modena, Lardo di Colonnata, Mortadella Bologna, and Salame Felino, alongside several protected designation of origin (PDO) products, including Prosciutto di Parma, Prosciutto di San Daniele, and Soppressata di Calabria. These products represent Italian gastronomic excellence, contributing to its global reputation for artisanal food while supporting local economies and exports. However, in recent decades, dietary habits and lifestyles in Italy have undergone notable changes. The rise of take-away and street food, along with increased tourism and the growth of multicultural communities, has driven a growing demand for ethnic food imports. Studies indicate that interest in ethnic food is accelerating in Western societies [63], including Italy [64]. Ethnic food consumption in Italy has increased significantly, with sales rising by 93% since 2007 [64]. Among ethnic food consumers, 75% purchase these products from shops and supermarkets. Ethnic foods are commonly found in large retailers, small grocery stores (often run by foreign nationals or Italians), fair trade outlets, organic stores, or online platforms. The most popular ethnic food products in Italy come from China/Japan, followed by Mexico/South America, the Middle East, Southeast Asia, Africa, and Eastern Europe [64].

Illegal imports have frequently been identified or suspected as the primary cause of transboundary animal disease outbreaks [37], with ASF serving as a noticeable example. ASF has repeatedly been introduced into disease-free areas through illegal imports of swine meat products by tourists, for commercial purposes, or via improper disposal of waste from ships or planes originating from ASF-affected regions [37]. Such incidents were suspected to be the cause of outbreaks in Spain during the 1960s [65], in Cuba and Brazil during the 1970s [66, 67], and more recently in Mauritius in 2007 [68] and the Caucasus in 2007 and 2008 [8, 69]. Similarly, human-mediated activities were strongly suspected in mainland Italy [24].

The incidental detection of an illegally imported non-EU animal product PCR-positive for ASFv, identified during routine regulatory compliance inspections, emphasized the need for greater vigilance regarding pork-based imports. It also underscored the importance of strengthening border controls and traceability systems, confirming the role of food commodities as a potential pathway for transboundary disease introduction. This unexpected finding subsequently prompted the establishment of two dedicated monitoring programs: one targeting the illegal domestic trade of wild boar meat and meat products, and the other focusing on ethnic food imports.

The nationwide sampling activities, conducted in accordance with both the monitoring programs, required extensive coordination among regional authorities, local services, and the NRL to execute the controls efficiently within a limited timeframe. To ensure the reliability and diagnostic capacity of the system established by the NRL, two key measures were implemented. First, the real-time PCR assay was validated on food matrices, in accordance with international standards, to guarantee reliable ASFv detection across heterogeneous sample types. Second, both in vitro and in vivo studies—the latter recognized as the best method for assessing ASFv infectivity [38]—were conducted to evaluate the infectivity of real-time PCR-positive samples.

A limitation of this study lies in the absence of a comprehensive national overview of the illegal importation of ethnic food, arising from two complementary factors. First, reporting from the regions to the Ministry of Health was incomplete, preventing an accurate quantification of the total number of inspections carried out by the competent authorities and of all irregular and confiscated products. Second, the samples submitted to the NRL for ASFv real-time PCR testing represented only a subset of confiscated items, as only those containing swine-derived ingredients—either indicated on the label or confirmed through genetic analysis—and lacking clear evidence of inactivation treatment were forwarded for testing. Anyway, both monitoring programs highlighted the significant risk associated with the illegal domestic trade of wild boar meat and meat products and the illegal importation of ethnic foods. Most samples (87%) came from the ethnic food monitoring program and were seized due to missing or unclear labeling; for example, some products omitted swine meat from the ingredient list, but displayed pig images on the packaging. In such cases, products were submitted to swine-specific genetic marker analysis. In this context of insecurity, when uncertainty remained regarding inactivation treatments, the competent authority, applying the precautionary principle, required ASFv real-time PCR testing, even for products that were likely subjected to thermal processing (e.g., wurstel, canned meat, and cooked meat product).

Within this context of illegality, molecular analysis performed by the NRL revealed that all samples derived from unauthorized domestic trade tested negative for ASFv, whereas a significant proportion (26%) of suid-derived products confiscated from illegal imports tested positive by real-time PCR. Regional distribution of ASFv-PCR-positive samples detected during the monitoring program for illegally imported ethnic food revealed that certain points of sale were sampled more over the monitoring period, suggesting a recurring involvement in the illegal trade of contaminated products.

Molecular characterization, aimed at determining the genotypes present in the contaminated meat and meat products, was attempted. The partial molecular characterization of virus vP72, conducted by PCR using primers p72-D and p72-U which amplify a 478 bp C-terminal region of the p72 gene [70], represent the primary molecular approach used for genotyping ASFvs and for inferring broad epidemiological relationships between the 24 known genotypes [52, 70]. However, this approach did not yield results in any samples most likely due to the extensive degradation of genetic material in ultraprocessed products, which resulted in poor DNA quality, or due to the presence of PCR inhibitors such as food additives (data not shown). To overcome this limitation, an alternative approach targeting shorter fragments of vP72 was employed and has proven useful to characterize the processed products. Specifically, the primer set PPA1–PPA2, which amplifies a 257 bp fragment, was used as previously described by Agüero et al. [49]. In addition, the CVR within the B602L gene was analyzed for all samples to provide further discriminatory power [50, 51]. The phylogenetic analysis established that all the analyzed samples were placed in the Vp72 Genotypes I and II. Alignment and translation of sequences obtained revealed the SNPs presence in seven samples. Several studies have reaffirmed the epidemiological utility of the CVR genome region within B602L gene for distinguishing between geographically and temporally constrained genotype [70]. Particularly, analysis of the amino acid tetramer repeat sequences within the CVR of seven samples assigned the Genotype I viruses to the Tet-10a variant group, clustering with strains previously reported in southern China during 2019–2020 [61] and never described in Italy during the endemicity of ASFv in Sardinia. In contrast, CVR sequences from Genotype II viruses in the same samples yielded a different amplicon. These grouped within Genotype II, Subgroup I, together with the reference strain Georgia 2007/1 [52]. They did not represent a novel subgroup but instead corresponded to the previously described CVR Genotype II-variant I (CVR1), characterized by the BNDBNDBNAA tetrameric sequence. Overall, this molecular approach revealed the codetection of multiple ASFv genotypes, specifically Genotypes I and II, with some samples showing the simultaneous presence of both. These findings suggested that the contaminated products likely originated from countries where both genotypes are currently cocirculating. This hypothesis was further supported by product labeling, often written in Chinese characters, and by phylogenetic analysis, which demonstrated a high degree of genetic similarity with ASFv strains previously reported in China.

The illegally imported ethnic foods may have been contaminated either through the use of infected animals or during processing. This raises serious concerns about the quality of raw meat and the adequacy of production processes, particularly with respect to hygiene, biosafety, and compliance with sanitary standards. A similar issue was recently reported in Indonesia, where ASFv was detected in pork products imported from China [71], confirming that this is not an isolated phenomenon but part of a broader global trend affecting multiple regions.

According to the NOVA classification, foods are divided into four groups, one of which is ultraprocessed products (Group 4), including snacks, ready meals, and other formulations largely composed of food-derived substances [72]. These products rely heavily on additives that replicate or intensify sensory qualities, making them convenient (ready-to-consume and long-lasting), highly palatable, and profitable due to low-cost ingredients and extended shelf life. Such features, combined with aggressive marketing, explain why ultraprocessed foods is rapidly increasing especially in middle-income countries, even where culinary traditions based on freshly prepared meals have historically been preserved, with annual growth rates reaching up to 10% [73]. In the present study, the majority of ASFv-PCR-positive samples fell into the category of ultraprocessed products, including those consisting of raw meat prepared with culinary ingredients, food additives, and processed foods. Only one product belonged to the processed food category (1%; Group 3). Interestingly, virus isolation performed on all real-time PCR-positive samples yielded negative results, suggesting that ASFv was likely inactivated during the processing and transformation steps typical of these products. Unexpectedly, even raw and dried meat samples (NOVA Groups 4 and 3, respectively), which would normally be expected to pose a higher risk, also tested negative. It is plausible that processes such as drying, or the addition of culinary ingredients, additives, and processed components such as soy sauce contributed to ASFv inactivation. Overall, these findings appear reassuring regarding the low potential risk of ASFv transboundary transmission by highly processed pork products; however, they should be interpreted with caution, as they are based on a limited number of samples and specific, not fully characterized, processing conditions. The variability of production practices and their impact on virus inactivation underline the difficulty of accurately assessing ASFv contamination risk in food products and highlight the need for further studies to elucidate the relationship between food processing practices and virus inactivation.

To strengthen the robustness of in vitro findings, an in vivo trial was conducted using a subset of the food products previously tested by real-time PCR and virus isolation. Due to sample depletion during earlier analyses, it was not possible to include certain samples, including the dried meat sample. From the remaining contaminated products, 30 were selected to represent different categories of foodstuffs. The number of tested samples was deliberately limited, as only four pigs were available (divided into two groups) in accordance with the “reduction” principle [74], and administering a larger quantity of products could have caused gastrointestinal complications. This approach was intended to maintain a careful balance between scientific rigor and ethical responsibility. The in vivo trial yielded negative results. All pigs fed with ASFv-PCR-positive products showed no clinical signs or fever, and postmortem examinations revealed no lesions indicative of ASFv infection. Blood and tissue samples collected at necropsy, as well as sera obtained prior to culling, tested negative for ASFv by real-time PCR and ELISA, respectively, confirming that the animals neither became infected nor developed antibodies. Together with the results of virus isolation, these findings indicated that, despite the ASFv genome being detectable by real-time PCR, the virus present in the tested food products was not infectious.

Nevertheless, the detection of illegally imported meat products PCR-positive for ASFv particularly raw meats, raises serious concern about the potential introduction of new ASFv genotypes into Italy and the risk of spreading the disease to currently unaffected areas. Indeed, it remains plausible that, contaminated products containing infectious virus could reach the Italian market and come into contact with susceptible animals, with potentially severe consequences. This risk cannot be entirely ruled out, underscoring the importance of continued vigilance and stringent control measures to mitigate this tangible threat.

## 5. Conclusion

The intensified control measures implemented in Italy to ensure compliance of meat and meat products derived from wild boars and domestic pigs with established trade regulations revealed significant irregularities in domestic trade practices, as well as in the importation and labeling of ethnic food products. These findings highlight the existence of illegal activities that undermine the integrity of biosecurity protocols and exacerbate the risk of ASFv transmission. Importantly, targeting commercial distributors to identify illegally imported products that do not comply with traceability standards has proven particularly effective, while also revealing gaps in upstream surveillance systems. Italy's comprehensive and multidisciplinary approach, combining clearly defined sampling objectives, advanced molecular diagnostics, virus isolation, and in vivo testing, has proven crucial for managing the ASF threat linked to transboundary transmission through illegal importation and for accurately assessing the real risk posed by the introduction of highly processed pork products contaminated with ASFv. These results contribute to the global effort to control ASF and protect the swine industry from significant economic losses. Ongoing vigilance, coupled with refined laboratory techniques and coordinated nationwide efforts, is essential to safeguard the swine industry and prevent future outbreaks. Further research and international collaboration will be vital in enhancing these measures and developing new strategies to combat ASF. Only through a unified and proactive approach can the swine industry be effectively protected, ensuring both animal health and food security remain safeguarded.

## Data Availability

The data that support the findings of this study are available from the corresponding author upon reasonable request.

## Supporting Information

Supporting Information Table S1: Description of ASFv-positive suid-meat and derived products detected by real-time PCR and subsequently used for virus isolation and in vivo experimental trials. Reported information includes: sample ID, date of collection, product category, product type, NOVA food processing category, evidence of inactivation treatment or uncertainty, origin of sampling (local trade/import), samples used for viral isolation and experimental trials, sequencing outcome, ASFv genotype, and accession number for B646L/Vp72 and B602L/CVR.
